# Proteinuria in COVID-19: prevalence, characterization and prognostic role

**DOI:** 10.1007/s40620-020-00931-w

**Published:** 2021-01-23

**Authors:** Justine Huart, Antoine Bouquegneau, Laurence Lutteri, Pauline Erpicum, Stéphanie Grosch, Guillaume Résimont, Patricia Wiesen, Christophe Bovy, Jean-Marie Krzesinski, Marie Thys, Bernard Lambermont, Benoît Misset, Hans Pottel, Christophe Mariat, Etienne Cavalier, Stéphane Burtey, François Jouret, Pierre Delanaye

**Affiliations:** 1 Department of Nephrology-Dialysis-Transplantation, Service de Dialyse, CHU Sart Tilman, University of Liège (CHU ULiege), 4000 Liège, Belgium; 2 Groupe Interdisciplinaire de Géno-protéomique Appliquée, Cardiovascular Sciences, University of Liège, Liège, Belgium; 3 Department of Clinical Chemistry, CHU Sart Tilman, University of Liège (CHU ULiege), Liège, Belgium; 4 Department of Intensive Care, CHU Sart Tilman, University of Liège (CHU ULiege), Liège, Belgium; 5 Department of Medico-Economic Information, CHU Sart Tilman, University of Liège (CHU ULiege), Liège, Belgium; 6 Department of Public Health and Primary Care, KU Leuven Campus Kulak Kortrijk, Kortrijk, Belgium; 7 Nephrology, Dialysis and Renal Transplantation Department, Hôpital Nord, CHU de Saint-Etienne, Jean Monnet University, COMUE Université de Lyon, Lyon, France; 8 Center of Nephrology and Renal Transplantation, Public Assistance of the Hospitals of Marseille, Marseille, France; 9 Aix-Marseille University, INSERM, INRA, C2VN, Marseille, France; 10 Department of Nephrology-Dialysis-Apheresis, Hopital Universitaire Caremeau, Nimes, France

**Keywords:** Proteinuria, COVID-19, α_1_-microglobulin, Tubular proteinuria

## Abstract

**Background:**

Proteinuria has been commonly reported in patients with COVID-19. However, only dipstick tests have been frequently used thus far. Here, the quantification and characterization of proteinuria were investigated and their association with mortality was assessed.

**Methods:**

This retrospective, observational, single center study included 153 patients, hospitalized with COVID-19 between March 28th and April 30th, 2020, in whom total proteinuria and urinary α_1_-microglobulin (a marker of tubular injury) were measured. Association with mortality was evaluated, with a follow-up until May 7th, 2020.

**Results:**

According to the Kidney Disease Improving Global Outcomes staging, 14% (n = 21) of the patients had category 1 proteinuria (< 150 mg/g of urine creatinine), 42% (n = 64) had category 2 (between 150 and 500 mg/g) and 44% (n = 68) had category 3 proteinuria (over 500 mg/g). Urine α_1_-microglobulin concentration was higher than 15 mg/g in 89% of patients. After a median follow-up of 27 [14;30] days, the mortality rate reached 18%. Total proteinuria and urinary α_1_-microglobulin were associated with mortality in unadjusted and adjusted models. This association was stronger in subgroups of patients with normal renal function and without a urinary catheter.

**Conclusions:**

Proteinuria is frequent in patients with COVID-19. Its characterization suggests a tubular origin, with increased urinary α_1_-microglobulin. Tubular proteinuria was associated with mortality in COVID-19 in our restropective, observational study.

**Supplementary Information:**

The online version contains supplementary material available at 10.1007/s40620-020-00931-w.

## Introduction

The SARS-CoV-2 (severe acute respiratory syndrome coronavirus 2) causes “coronavirus disease 2019” (COVID-19) [1, 2] which is characterized by diffuse alveolar damage leading to an acute respiratory distress syndrome [3]. Other organs may also be affected [2–4]. Cheng et al. [2] early described a high prevalence of proteinuria (43.9%) and hematuria (26.7%) in a cohort of 442 patients in Wuhan, China [2]. These data suggest specific kidney damage caused by SARS-CoV-2, although it remains unclear whether the virus is present in the kidney or urine, or not [5, 6]. Since this first publication, measurement of proteinuria has been recommended in our institution, using quantitative measurements and characterization of proteinuria. Dipstick methods of assessing proteinuria have well-established limitations [7]. In the present paper, we aimed to characterize the prevalence and type of proteinuria observed in patients with COVID-19, and also to assess the prognostic importance of proteinuria in COVID-19.

## Patients and methods

This was an observational retrospective single-center study. Clinical and biological variables were extracted from the computer-based medical records. All data were anonymized and the study was approved by the Ethics Committee of Liège Academic Hospital.

*Inclusion criteria* All patients older than 18 years admitted to Liège Academic Hospital between March 28th and April 30th, 2020 with a positive COVID-19 test were eligible. A positive test was obtained by reverse transcriptase polymerase chain reaction (RT-PCR, Cobas SARS CoV-2 Test with Cobas 8800) via nasal swab or antigen testing. Only one patient had positive antigen without RT-PCR. Only patients with at least one complete urine analysis (red blood cell (RBC) count, total proteinuria and α_1_-microglobulin) were considered for the present analysis. Quantification of urinary β_2_-microglobulin was also available in a subset of patients. Patients on renal replacement therapy (i.e. renal transplantation or chronic dialysis) were excluded.

*Urine samples* The day the urine was collected was considered Day 0 (D0). A second urine analysis was performed in some patients on Day 7 (D7). Total proteinuria (expressed in mg/g of urine creatinine) was measured on a random spot urine sample, with Abbott Alinity instrument, whereas urine α_1_-microglobulin and β_2_-microglobulin were determined with the Siemens Dimension Vista instrument. Proteinuria was staged according to the KDIGO (Kidney Disease Improving Global Outcomes) categorization system [8]: normal or category 1 (< 150 mg/g), category 2 (between 150 and 500 mg/g), category 3 (over 500 mg/g). Urine α_1_-microglobulin was expressed in mg/g of urine creatinine. Tertiles and a fixed threshold were considered (> 15 mg/g based on the current literature [9, 10]). Urine β_2_-microglobulin was expressed in mg/L. Tertiles and a result above 0.19 mg/L was considered abnormal [11]. The number of RBCs in the urine was automatically evaluated by the Sedimax automate (positive if > 10 RBC per field). Because proteinuria and hematuria can be impacted by urinary catheter (UC), analyses were repeated in subgroups without UC at D0.

### Clinical, biological and radiological parameters

The following variables were considered: age, weight, height, body mass index (BMI), history of hypertension (based on medical records and/or the presence of antihypertensive medications at admission), history of diabetes (based on medical records and/or the presence of specific therapy at admission), active cancer, active smoking, and history of chronic kidney disease (CKD) (based on medical records, not on biological data). Biological data of interest were considered at the closest time of measurement to D0 within a maximum of 48 h. All biological data were generated from one single laboratory (Unilab, CHU de Liège) accredited for ISO 15,189 Guideline. The following variables were collected: C-reactive protein (CRP), procalcitonin, serum creatinine, lactate dehydrogenase (LDH), albumin, sodium, potassium, total calcium, bicarbonate concentrations (Abbott Alinity instrument), leukocytes, lymphocytes, platelet counts, hemoglobin (Sysmex SE-9000 Hematology analyzer), and D-Dimer (Innovance D-Dimer kit on the Siemens CS5100 automate).

A clear distinction between CKD and acute kidney injury (AKI) at admission was not always possible. Therefore, we used the term “decreased kidney function”, based on the CKD-Epidemiology equation and using an age-calibrated definition: estimated glomerular filtration rate (eGFR) below 75, 60 or 45 mL/min/1.73 m^2^ for patients younger than 40 years, between 40 and 65 years or older than 65 years, respectively [12, 13]. Because proteinuria and hematuria can be influenced by CKD status and/or AKI, analyses were repeated in subgroups without decreased eGFR at D0. AKI during hospitalization (i.e. after D0) was also considered, and based on the KDIGO definition, the first serum creatinine concentration at admission was considered the baseline [14]. Statistical analyses were repeated in subgroups with and without AKI during hospitalization.

Thorax CT-scanner staging was used to assess the radiographic severity of COVID-19 pneumonia (percentage of the lungs involved) as reported by radiologists in medical records (except for two patients): < 10%, between 10 and 50%, and > 50%.

Severe cases on D0 were defined according to the guidelines of the Chinese National Health Commission: (1) respiratory rate > 30 breaths/min, (2) oxygen saturation < 93%, or (3) PaO_2_/FiO_2_ ratio < 300 mmHg [2].

Mortality was checked for all patients in the medical records and/or confirmed by a phone call to the general practitioners until May 7th, 2020.

### Statistical analyses

Data are expressed as mean ± standard deviation (SD) when the distribution was normal and as median with quartiles when not. Normality was assessed by the Shapiro–Wilk test. Comparison of two independent groups was performed using Mann–Whitney U test or Chi square test for continuous and categorical variables, respectively. Kruskal–Wallis test with post hoc test according to Dunn and exact Chi^2^ test (with Bonferroni correction) were performed to compare more than two groups for continuous variables and categorical variables, respectively. Univariate survival analysis (Kaplan–Meier) was done with categorical urine variables (according to KDIGO for proteinuria, to tertiles for urine α_1_-microglobulin and β_2_-microglobulin, and more than 10 RBCs per field for hematuria) as strata. Cox proportional hazard regression modeling using the backward selection procedure was performed to study the risk of mortality associated with all variables available in Table 1. Proteinuria, urine α_1_-microglobulin, hematuria, and the presence of UC were considered categorical variables. These urine variables were studied in non-adjusted models and in different models adjusted for other covariates at D0 that were significantly associated with mortality in the unadjusted model. All statistics were performed with MedCalc statistical software (Medcalc, Mariakerke, Belgium).

**Table 1:** Clinical and biological characteristics of the cohort at D0

Clinical	
Age (years) (n = 153) < 40 years (%) [40–65] years > 65 years (%)	70 [58;81] 7 35 59
Women (n = 153) (%)	39
Weight (n = 130) (kg)	80 ± 17
Height (n = 125) (m)	1.70 [1.62;1.78]
Body Mass index (n = 120) (kg/m^2^)	
< 18.5 (%) [18.5–25] (%) [25–30] (%) ≥ 30 (%)	28 [24;31] 3 27 43 27
Medical history (n = 153)	
Hypertension (%) Diabetes (%) Chronic kidney disease (%) Active cancer (%) Active smoking (%)	59 43 17 10 5
Intensive Care Unit (%)	22
Severe case (%)	82
Thoracic CT-Scanner staging (n = 151) (%)	
Normal Minor (< 10%) Mild (10–50%) Severe (> 50%)	17 7 61 16
Biological (D0)	
C-reactive protein (n = 153) (mg/L) C-reactive protein > 5 mg/L	81 [38;155] 97%
Leukocytes (n = 153) (/mm^3^) >10,100/mm^3^ (%) <4600/mm^3^ (%)	6180 [4650;9060] 19 24
Lymphocytes (n = 151) (/mm^3^)	860 [655;1155]
Hemoglobin (n = 153) (g/dL)	12.3 [11.2;13.6]
Platelets (n = 153) (× 1000/mm^3^)	230 [165;301]
Lactate Dehydrogenase (n = 153) (U/L) > 220 (%)	342 [259;443] 87
D-Dimer (n = 141) (µg/L) > 500 (%)	1009 [715;1878] 84
Albumin (n = 148) (g/L) < 32 (%)	34 ± 5 30
Potassium (n = 153) (mmol/L) > 5.1 (%) < 2.5 (%)	4.10 ± 0.47 2 9
Sodium (n = 153) (mmol/L) < 136 (%)	139 [137;142] 14
Calcium (n = 153) (mmol/L) < 2.2 (%)	2.10 [2.01;2.20] 72
Bicarbonate (n = 145) (mmol/L) < 22.1 (%) > 31 (%)	25.5 [23.1;27.6] 17 7

## Results

### Characteristics of the population

The study cohort included 153 patients (flowchart of patient inclusion in Figure S1). The median time period between admission and D0 was 3 [2, 5] days. The patients excluded from the analysis because of the lack of urine samples (n = 72) were more frequently women and had a higher and earlier mortality rate (Table S1). The median age of our cohort was 70 [58-81] years, and 39% were women (Table 1). COVID-19 patients were characterized by high serum CRP and LDH concentrations and low lymphocyte number (Table 1). Eighty-two  % of patients were staged as with severe pneumonia. After D0, 29 patients (19%) developed AKI (7 reached stage 2 and 5 reached stage 3). Twenty-three (15%) patients died during hospitalization and 4 deaths occurred after the patients left the hospital, leading to a mortality rate of 18% during the study period. At the end of the follow-up, 17 patients were still hospitalized.

### Renal parameters

On D0, serum creatinine concentration was higher than normal in 27% of patients. A decreased renal function on D0 was observed in 24% of patients.

### Proteinuria

The median proteinuria in our cohort was 455 [238;834] mg/g at D0. Fourteen percent (n = 21) of the patients had category 1 proteinuria, 42% (n = 64) had category 2 and 44% (n = 68) had category 3 proteinuria. Two patients had very high proteinuria (over 3500 mg/g). Clinical and biological characteristics according to proteinuria categories are shown in Table 2. Patients in higher proteinuria categories were older, and more severely affected (more patients in ICU and higher concentrations of CRP, as examples).

**Table 2:** Clinical and biological characteristics of the patients at D0 according to proteinuria staging at D0 (n = 153)

	Stage 1 proteinuria <150 mg/g (n = 21)	Stage 2 150-500 mg/g (n = 64)	Stage 3 >500 mg/g (n = 68)	Kruskal–Wallis test (or exact Chi^2^ test)
*Clinical*				
Age (years)	61 [55;79]	66 [58;79]	75 [64;84]	0.02
Women (%)	43	42	34	ns
Weight (kg)	77 [65;92]	82 [69;95]	76 [67;91]	ns
Height (m)	1.67 [1.61;1.77]	1.71 [1.63;1.78]	1.70 [1.65;1.79]	ns
Body Mass index (kg/m^2^)	27 [25;29]	28 [25;31]	27 [23;29]	ns
*Medical history*				
Hypertension (%) Diabetes (%) Chronic kidney disease (%) Active cancer (%) Active smoking (%)	48 33 5 5 0	58 27 16 13 6	63 28 22 10 4	ns ns ns ns ns
Intensive Care Unit (%)	5*	17	31	0.006
Severe cases (%)	48	84^$^	90^$^	0.0002
*Thoracic CT-Scanner staging*				
Normal Minor (< 10%) Mild (10–50%) Severe (> 50%)	30 10 55 5	11 10 65 14	13 4 59 24	ns ns ns 0.04
*Biological*				
Creatinine (D0)(mg/dL) eGFR (mL/min/1.73 m^2^)	0.87 [0.72;0.95] 85 [70;97]*	0.92 [0.72;1.2] 74 [59;92]	0.98 [0.82;1.33] 69 [51;85]	ns 0.02
C-reactive protein (mg/L)	49 [13;74]*	78 [26;148]*	118 [61;169]	0.0002
Leukocyte count (/mm^3^)	5720 [4170;7170]	6005 [4735;10,025]	6605 [4875;9150]	ns
Lymphocyte count (/mm^3^)	940 [763;1399]	910 [703;1248]	800 [543;1075]	0.04
Hemoglobin (g/dL)	13.2 [12.6;14.3]*	12.6 [11.1;13.8]	11.8 [11;13.1]	0.01
Platelets (x1,000/mm^3^)	248 [181;301]	240 [174;332]	201 [160;289]	ns
Lactate Dehydrogenase (U/L)	254 [224;290]*	328 [258;408]^$^	377 [285;555]	0.00005
D-Dimer (µg/L)	876 [371;1508]	952 [560;1609]	1202 [815;2945]	0.04
Albumin (g/L)	38 [33;41]*	35 [32;39]^*^	33 [33;41]	0.0002
Potassium (mmol/L)	4.16 [3.80;4.54]	4.12 [3.82;4.37]	4.04 [3.76;4.35]	ns
Sodium (mmol/L)	141 [139;143]	141 [137;141]	139 [136;143]	ns
Calcium (mmol/L)	2.23 [2.16;2.29]*	2.13 [2.05;2.23]*	2.03 [1.97;2.11]	0.000001
Bicarbonate (mmol/L)	26.3 [23.9;29.2]	24.7 [22.6;27.2]	25.7 [23.3;28.2]	ns

*Ns* not significant, *eGFR* estimated glomerular filtration rate

Dunn’s post hoc test p < 0.05: * proteinuria category 3, $ with proteinuria category 1

Chi^2^ test for categorical variables with Bonferroni correction: * with proteinuria category 3

Among the 153 patients, a pre-admission value of proteinuria was available for 51 patients, within a median preceding time of 383 [161;836] days. Among the 32 patients with prior abnormal proteinuria, only 2 had normal proteinuria at D0. Conversely, among the 19 patients with prior normal proteinuria, only 4 remained within the normal range during the study period. One of the two patients with very high proteinuria during COVID-19 had normal proteinuria documented 57 days before D0. Limiting analysis to the 112 patients with less than 10 RBCs per field, abnormal proteinuria was found in 81% of patients. Among the 114 patients without UC, 82% had proteinuria (38% with category 3). Among the 122 patients without decreased eGFR on D0, 83% had abnormal proteinuria (43% with category 3). Among the 124 patients who did not develop AKI after D0, 84% had abnormal proteinuria (38% with category 3).

The median urinary concentrations of α_1_-microglobulin and β_2_-microglobulin (n = 94) were 54 [27;122] mg/g and 2.65 [0.40;14.15] mg/L, respectively. α_1_-microglobulin concentration was higher than 15 mg/g in 89% of patients. Urine β_2_-microglobulin was higher than 0.19 mg/L in 85% of patients. Among the 114 patients without UC, urine α_1_-microglobulin over 15 mg/g and urine β_2_-microglobulin over 0.19 mg/L were found in 86% and 84% of the patients, respectively. Among the 122 patients without decreased eGFR on D0, 87% and 83% had α_1_-microglobulin and β_2_-microglobulin concentrations over 15 mg/g and 0.19 mg/L, respectively. Among the 124 patients who did not develop AKI after D0, 87% and 82% had α_1_-microglobulin and β_2_-microglobulin concentrations over 15 mg/g and 0.19 mg/L, respectively.

Table 3 describes the clinical and biological characteristics of the patients according to tertiles of urine α_1_-microglobulin and Table S2 describes the clinical and biological characteristics of the patients according to tertiles of urine β_2_-microglobulin. As for categories of total proteinuria, higher tertiles of α_1_-microglobulin and β_2_-microglobulin are associated with older age and factors of severity.

**Table 3:** Clinical and biological characteristics of the patients at D0 according to tertiles of urinary α_1_-microglobulin at D0

	Tertile 1 < 32 mg/g (n = 51)	Tertile 2 32–86 mg/g (n = 50)	Tertile 3 > 86 mg/g (n = 52)	Kruskal–Wallis test (or exact Chi^2^ test)
*Clinical*				
Age (years)	66 [57;80]*	65 [58;73]*	77 [70;85]	0.005
Women (%)	49	34	33	ns
Weight (kg)	79 [65;94]	83 [69;93]	75 [68;90]	ns
Height (m)	1.68 [1.61;1.79]	1.74 [1.67;1.79]	1.70 [1.65;1.76]	ns
Body Mass index (kg/m^2^)	28 [25;31]	28 [23;30]	27 [23;29]	ns
*Medical history*				
Hypertension (%) Diabetes (%) Chronic kidney disease (%) Active cancer (%) Active smoking(%)	57 29 4* 8 6	62 18 8* 10 2	58 37 38 6 13	ns ns < 0.0001 ns ns
Intensive Care Unit (%)	14	24	27	ns
Severe cases (%)	69*	86	90	0.004
*Biological*				
Creatinine (D0)(mg/dL) eGFR (mL/min/1.73 m^2^)	0.87 [0.70;0.99]* 82 [61;95]*	0.91 [0.76;1.06] 82 [61;93]*	1.04 [0.86;1.49] 61 [45;83]	0.007 0.002
C-reactive protein (mg/L)	61 [15;108]	135 [54;216]^$^	95 [47;147]^$^	0.0001
Leukocyte count (/mm^3^)	5940 [4260;5940]	6720 [5170;10,100]	6110 [4630;8615]	ns
Lymphocyte count (/mm^3^)	925 [720;1350]	850 [630;1050]	820 [535;1095]	ns
Hemoglobin (g/dL)	12.9 [12.0;14.0]*	12.6 [11.6;13.7]*	11.3 [10.5;13.0]	0.0004
Platelets (× 1000/mm^3^)	248 [179;331]*	247 [189;320]*	180 [140;252]	0.0007
Lactate Dehydrogenase (U/L)	288 [234;382]	372 [274;531]^$^	361 [277;446]^$^	0.007
D-Dimer (µg/L)	972 [564;1590]	1091 [789;3090]	1050 [776;2208]	ns
Albumin (g/L)	37 [33;40]	33 [31;37]^$^	33 [29;36]^$^	0.0002
Potassium (mmol/L)	4.06 [3.76;4.40]	4.09 [3.91;4.30]	4.14 [3.72;4.48]	ns
Sodium (mmol/L)	140 [138;143]	138 [136;140]	140 [137;143]	0.04
Calcium (mmol/L)	2.18 [2.10;2.29]	2.07 [2.00;2.16]^$^	2.07 [1.97;2.12]^$^	0.000004
Bicarbonate (mmol/L)	25.9 [23.2;27.5]	24.5 [22.5;26.7]	26.1 [23.6;28.9]	ns

*Ns* not significant, *eGFR* estimated glomerular filtration rate

Dunn’s post hoc test p < 0.05: * proteinuria category stage 3, $ with proteinuria category 1

Chi^2^ test for categorical variables with Bonferroni correction: * with proteinuria category 3

### Hematuria

The prevalence of hematuria in the 153-patient cohort was 26%. In patients without UC (n = 114), the prevalence of hematuria was 13%.

### Factors at D0 associated with mortality

Mortality was assessed for all patients on May 7th, 2020 with a median follow-up of 27 [14, 30] days, D0 being the reference date. During the follow-up, 27 patients died (18%). The median time period between D0 and date of death was 7 [4, 12] days.

Comparison of variables at D0 between patients who died *versus* survived is shown in Table S3. The patients who died were older, more frequently men and more frequently had a history of CKD and known active cancer. They had lower eGFR, lower platelet counts and higher CRP concentrations. For urine parameters, proteinuria, urine α_1_-microglobulin, urine β_2_-microglobulin, number of RBCs per field and UC were also significantly higher in deceased patients compared to surviving patients. Urine analyses were also performed in sub-groups and showed similar results (Table S4).

**Fig. 1:**
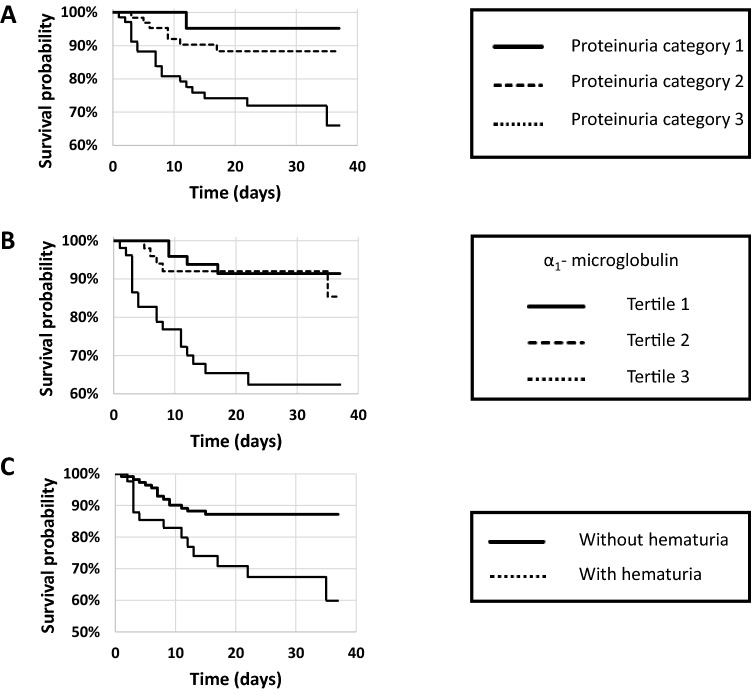
Survival curve according to proteinuria categorization (**a**), urine α_1_-microglobulin (**b**) or presence of hematuria (**c**)

In the univariate Cox proportional hazards regression analysis, the following parameters were associated with mortality: age (HR 1.03 95% CI 1.00–1.06, p = 0.03), male gender (HR 2.94 95% CI 1.22–7.69, p = 0.02), history of CKD (HR 2.35 95% CI 1.03–5.38, p = 0.04), active cancer (HR 2.95 95% CI 1.19–7.34, p = 0.02), eGFR (for 10 unit decrease: HR 1.16 95% CI 1.01–1.34, p = 0.03), CRP (for 10 unit increase: HR 1.04 95% CI 1.00–1.08, p = 0.04), platelet counts (for 10,000 unit decrease: HR 1.01 95% CI 1.00–1.01, p = 0.004), category 3 proteinuria (*versus* category 2: HR 2.83 95% CI 1.19-6.72, p = 0.02), urine α_1_-microglobulin (tertiles 3 *versus* 1: HR 5.41 95% CI 1.83–16.00, p = 0.002 and tertiles 3 *versus* 2: HR 4.40 95% CI 1.63-11.90, p = 0.004), hematuria (HR 2.82 95% CI 1.32–6.01, p = 0.007) and bladder catheterization (HR 4.18 95% CI 1.95–8.95, p = 0.0002). Kaplan–Meier survival curves for proteinuria categories, urine α_1_-microglobulin tertiles and hematuria (RBCs more than 10 per field) as strata are shown in Fig. 1. High tertiles of proteinuria and urine α_1_-microglobulin and hematuria were associated with lower survival (logrank test, p = 0.008, 0.0001 and 0.005, respectively). Focusing on urine variables, multivariable Cox adjusted analyses are shown in Table 4: category of proteinuria was not associated with mortality in the adjusted models, whereas urine α_1_-microglobulin remained associated with mortality in the adjusted models, except when the variable UC was included. Hematuria was associated with mortality in the adjusted models. However, such association was not observed in the subgroup of patients without UC. The presence of UC was highly predictive of mortality in all models. In patients with normal eGFR on D0, proteinuria and urine α_1_-microglobulin were still associated with mortality in the fully-adjusted model. The same results were observed in the sub-group without UC at D0. Forcing variables like score severity and hospitalization in ICU at D0 in the Cox models did not modify the results.

**Table 4:** Association between urine variables (proteinuria, α_1_-microglobulin, hematuria and urine catheter) and mortality in unadjusted and adjusted models in the whole cohort and subgroups

	Unadjusted	Model 1	Model 2	Model 3	Model 4
*The whole cohort* *N = 153*					
Proteinuria categorization (P)	P+	Age+ Women−	Age+ Women-	Age+ Women− CRP+ Platelets−	Age+ Platelets− UC+
Tertile of urine α_1_-microglobulin (Tα)	Tα+	Tα+ Women−	Tα+ Women- Cancer+	Tα+ Age+ Women− CRP+	Age + Platelets− UC+
Hematuria (Ht)	Ht+	Ht+ Age+ Women−	Ht+ Age+ Women− Cancer+	Age+ Women− CRP+ Platelets−	
Urinary catheter (UC)	UC+	UC+ Age+ Women−	UC+ Age+ Women−	UC+ Age+ Platelets−	
*Cohort with normal eGFR at D0* *N = 122*					
Proteinuria categorization (P)	P+	P+	P+ Women- Cancer+	P+ Age+ Cancer+ CRP+	P+ Age+ Cancer+ CRP+ UC+
Tertile of urine α_1_-microglobulin (Tα)	Tα+	Tα+	Tα+ Cancer+	Tα+ Cancer+ CRP+	Tα + Cancer+ CRP+ Ht+
Hematuria (Ht)	Ht+	Ht+	Ht+ Cancer+	Ht+ Age+ Cancer+ CRP+	
Urinary catheter (UC)	UC+	UC+	UC+ Cancer+	UC+ Age+ Cancer+ CRP+	
*Cohort without urinary catheter* *N = 114*					
Proteinuria categorization (P)	P+	P+	P+	P+ CRP+ Platelets−	P+ CRP+ Platelets−
Tertile of urine α_1_-microglobulin (Tα)	Tα+	Tα+	Tα+	Tα+ eGFR− CRP+	Tα + Age+ CRP+ Ht+
Hematuria	Ns	Age+	Age+	CRP+	

Only significant variables are shown in the table. + if the association is positive and − if the association is negative (or protective)

*CRP* C-reactive protein, *eGFR* estimated glomerular filtration rate, Ht hematuria, *P* proteinuria, Ns not significant, Tα Tertile of urine α_1_-microglobulin, *UC* Urine catheter, *Model 1* adjusted for age and gender, *Model 2* Model 1 + adjusted for CKD history and active cancer, *Model 3* Model 2 + adjusted for eGFR, CRP and platelet counts, *Model 4* Model 3 + adjusted for hematuria and urinary catheter

### Follow-up results at day 7

Among the 153 patients, 76 left the hospital less than 7 days after D0. Among the 77 patients still hospitalized at D7, 48 had a second measurement of proteinuria. Among them, the median concentration of proteinuria and urine α_1_-microglobulin at D0 was 493 [307;929] mg/g and 76 [37;144] mg/g, and 280 [170;521] mg/g and 60 [34;125] mg/g at D7, respectively. The median decrease in proteinuria and α_1_-microglobulin concentration was − 178 [− 531;− 52] mg/g (relative decrease of 43%) and − 17 [− 56;32] mg/g (relative decrease of 21%), respectively. Excluding the patients who died during the study period (n = 8) and those who were still hospitalized on May 7th (n = 6), it leaves 34 patients who left the hospital alive with a median concentration of proteinuria and α_1_-microglobulin on D0 of 483 [302;1062] mg/g and 60 [33;138] mg/g, and of 203 [109;328] mg/g and 34 [14;84] mg/g on D7, respectively. The median decrease in proteinuria and α_1_-microglobulin concentration was − 256 [− 717;− 98] mg/g (relative decrease of 58%) and − 20 [− 91;− 3] mg/g (relative decrease of 43%), respectively. Among these patients, proteinuria categories 1, 2 and 3 were observed in 9%, 44% and 47% respectively on D0. The prevalence was 32%, 50% and 18%, respectively on D7. Among the 3 patients with category 1 on D0, 2 progressed to category 2 at D7. Among the 15 patients with category 2 on D0, 8 decreased to category 1, one progressed to category 3 and 6 remained at category 2 proteinuria on D7. Among the 16 patients with category 3 proteinuria on D0, 2 decreased to category 1, 9 to category 2 and 5 remained at category 3 on day 7.

## Discussion

In a retrospective analysis of a cohort of 153 hospitalized patients with COVID-19, we found a high prevalence (close to 80%) of abnormal proteinuria (including 43% of them with category 3 proteinuria). Characterization of the proteinuria showed a predominant pattern of tubular proteinuria, as depicted by concomitant high levels of urine α_1_-microglobulin and β_2_-microglobulin. Eighty-nine  % of patients had increased α_1_-microglobulin values. Interestingly, total proteinuria and α_1_-microglobulin concentrations were predictive of mortality in our cohort.

The high prevalence of proteinuria in patients with COVID-19 was not related to hematuria in our study. Indeed, excluding patients with hematuria (26%), abnormal proteinuria was still found in 81% of cases. Moreover, proteinuria was also observed in patients who did not develop AKI after D0. Whether proteinuria is directly caused by SARS-CoV-2 infection of the renal parenchyma cannot be proven, due to the design of our study. However, pre-admission values of proteinuria were available for 51 patients and only 4 out of 19 patients who had prior normal proteinuria also had normal values at D0. This suggests de novo proteinuria due to COVID-19. Cheng et al. [2] observed proteinuria in 43.9% of COVID-19 patients. The same group in another publication reported that proteinuria was present in 65.8% of 333 hospitalized patients [15]. Hirsch et al. [16] found a prevalence of 42.1%, by dipstick results, in 646 patients in New-York City.

The present study is one of the first studies that characterize and quantify proteinuria in patients hospitalized with COVID-19. The fact that we measured proteinuria instead of employing dipsticks may per se explain the higher prevalence of proteinuria in our cohort, especially because dipstick testing is more sensitive for albuminuria than for tubular proteinuria [7]. Our analysis further revealed that proteinuria is mainly of tubular origin. The underlying mechanisms remain unknown. The design of the current study did not allow us to formally distinguish acute tubular necrosis (ATN) caused by a septic condition before D0 or admission to hospital, or low-oxygen delivery to tissues from specific cytopathic lesions caused by SARS-CoV-2 itself [17]. Some preliminary results with kidney biopsies (including electronic microscopy) suggest direct virus-mediated tubular injury [18–20]. This hypothesis is reinforced by the preferential tubular expression of angiotensin converting enzyme 2 which is suspected to participate in cellular entry of the SARS-CoV-2 [4, 21, 22]. However, the presence of the virus in the kidney (and in urine) is still the subject of active debate and further studies are needed [18–20, 22–24]. In our study, it is interesting to note that there were far more patients with isolated proteinuria than patients with decreased eGFR at admission (20% at D0). Only a small number of patients were hospitalized in intensive care units at D0, and the occurrence of AKI after D0 was19%.

Fifteen percent of patients died during hospitalization, a prevalence comparable to data available in New York City [25], Northern Italy [26], and China [2]. Parameters at D0 associated with mortality in our cohort (age, sex, history of CKD, active cancer, lower eGFR and platelet counts, and higher CRP) have already been reported by others [27, 28]. Interestingly, proteinuria and even more so, uriary α_1_-microglobulin, seem to be associated with mortality. Urine catheterization was highly predictive of mortality, probably as a reflection of frailty of the patient and/or severity of the disease. Because urine catheterization itself can cause hematuria and thus contribute to “false” total proteinuria, it is legitimate to discard patients with UC at D0 from the survival analysis. In the sub-group without UC, the predictive value of proteinuria and urine α_1_-microglobulin was confirmed. Hematuria was not predictive of mortality per se. Cheng et al. [2] also found an association between kidney involvement, including proteinuria (by dipstick), and in-hospital death. Once again, the design of our study only allows to generate hypotheses to explain the higher mortality rate in patients with abnormal proteinuria (higher viral load and early multiple organ involvement [17, 29] or marker of ATN and/or AKI). Association is not causation, and the main cause of death in COVID-19 was respiratory failure, not kidney injury. In this context, and whatever the potential mechanisms, association between tubular proteinuria and mortality should be interpreted with caution. Residual confounding factors in the multivariable analysis remain possible.

The long-term renal consequences of COVID-19 are still unknown and will require follow-up studies. Pei et al. [15] observed that proteinuria often resolved (in 68.5% of the patients with prior proteinuria) within 3 weeks. Our preliminary data seem reassuring, but more distant follow-up of proteinuria is needed.

Our study has limitations. First, the study is single-center and retrospective. Second, all analyses were performed on D0, which was defined by the time of urine analysis. A median period of 3 days separates admission and D0, and our results must be interpreted accordingly. Significant differences between included and non-included patients (Table S1) are observed, essentially in terms of mortality and time to death. This bias is mainly due to a lack of urine collection in severely ill patients who died rapidly after admission. The analyses concerning mortality rate should also be considered with caution because of the small sample size. Tubular involvement suggested in our study does not exclude exceptional forms of glomerular involvement like collapsing glomerulopathy [30]. Data about urinary albumin were not available (not reimbursed by the Belgian health system) and mixed proteinuria cannot be excluded. Lastly, important variables like weight and height were not available for some patients.

In conclusion, a very high rate of tubular proteinuria is found in hospitalized COVID-19 patients. Abnormal proteinuria and/or elevated urine α_1_-microglobulin excretion were associated with mortality, in adjusted models. This is especially relevant in patients with normal eGFR at D0 and in patients without urinary catheters.

## Supplementary Information

Below is the link to the electronic supplementary material.

Supplementary material 1 (DOCX 55 kb)

## Data Availability

The data underlying this article will be shared on reasonable request to the corresponding author.

## References

[1] Zhu N, Zhang D, Wang W (2020). A novel coronavirus from patients with pneumonia in China, 2019. N Engl J Med.

[2] Cheng Y, Luo R, Wang K (2020). Kidney disease is associated with in-hospital death of patients with COVID-19. Kidney Int.

[3] Huang C, Wang Y, Li X (2020). Clinical features of patients infected with 2019 novel coronavirus in Wuhan, China. Lancet.

[4] Perico L, Benigni A, Remuzzi G (2020). Should covid-19 concern nephrologists? why and to what extent? The emerging impasse of angiotensin blockade. Nephron.

[5] Peng L, Liu J, Xu W (2020). SARS-CoV-2 can be detected in urine, blood, anal swabs, and oropharyngeal swabs specimens. J Med Virol.

[6] Wang W, Xu Y, Gao R (2020). Detection of SARS-CoV-2 in different types of clinical specimens. JAMA.

[7] Résimont G, Piéroni L, Bigot-Corbel E, Cavalier E, Delanaye P (2020). Urinary strips for protein assays: easy to do but difficult to interpret!. J Nephrol.

[8] Kidney Disease: Improving Global Outcomes (KDIGO) CKD Work Group (2013). KDIGO 2012 Clinical practice guideline for the evaluation and management of chronic kidney disease. Kidney Int Suppl.

[9] Yu H, Yanagisawa Y, Forbes MA, Cooper EH, Crockson RA, MacLennan IC (1983). Alpha-1-microglobulin: an indicator protein for renal tubular function. J Clin Pathol.

[10] Holzscheiter L, Beck C, Rutz S (2014). NGAL, L-FABP, and KIM-1 in comparison to established markers of renal dysfunction. Clin Chem Lab Med.

[11] Lammers M, Gentzer W, Reifferscheidt GSB (2002). Determination of beta2-microglobulin by a particle-enhanced immunonephelometric assay. Clin Chem.

[12] Delanaye P, Jager KJ, Bökenkamp A (2019). CKD: a call for an age-adapted definition. J Am Soc Nephrol.

[13] Delanaye P, Glassock RJ, Hans P, Rule AD (2016). An age-calibrated definition of chronic kidney disease: rationale and benefits. Clin Biochem Rev.

[14] KDIGO (2012). KDIGO clinical practice guideline for acute kidney injury. Kidney Int Suppl.

[15] Pei G, Zhang Z, Peng J (2020). Renal Involvement and Early Prognosis in Patients with COVID-19 Pneumonia. J Am Soc Nephrol.

[16] Hirsch JS, Ng JH, Ross DW (2020). Acute kidney injury in patients hospitalized with COVID-19. Kidney Int.

[17] Aleebrahim-dehkordi E, Reyhanian A, Saberianpour S, Hasanpour-dehkordi A (2020). Acute kidney injury in COVID-19; a review on current. J Nephropathol.

[18] Diao B, Feng Z, Wang C (2020). Human kidney is a target for novel severe acute respiratory syndrome coronavirus 2 (SARS-CoV-2) infection. medRxiv.

[19] Farkash EA, Wilson AM, Jentzen JM (2020). Ultrastructural evidence for direct renal infection with SARS-CoV-2. J Am Soc Nephrol.

[20] Su H, Yang M, Wan C (2020). Renal histopathological analysis of 26 postmortem findings of patients with COVID-19 in China. Kidney Int.

[21] Hoffmann M, Kleine-Weber H, Schroeder S (2020). SARS-CoV-2 cell entry depends on ACE2 and TMPRSS2 and is blocked by a clinically proven protease inhibitor. Cell.

[22] Batlle D, Soler MJ, Sparks MA (2020). Acute kidney injury in COVID-19: emerging evidence of a distinct pathophysiology. J Am Soc Nephrol.

[23] Gross O, Moerer O, Weber M, Huber TB, Scheithauer S (2020). COVID-19-associated nephritis: early warning for disease severity and complications?. Lancet.

[24] Roufosse C, Curtis E, Moran L (2020). Electron microscopic investigations in COVID-19: not all crowns are coronas. Kidney Int.

[25] Goyal P, Choi JJ, Pinheiro LC (2020). Clinical characteristics of Covid-19 in New York City. N Engl J Med.

[26] Inciardi RM, Adamo M, Lupi L (2020). Characteristics and outcomes of patients hospitalized for COVID-19 and cardiac disease in Northern Italy. Eur Heart J.

[27] Bhargava A, Fukushima EA, Levine M, Zhao W, Tanveer F, Susanna M, Szpunar LS (2020). Predictors for severe COVID-19 infection. Clin Infect Dis.

[28] Docherty AB, Harrison EM, Green CA (2020). Features of 20 133 UK patients in hospital with covid-19 using the ISARIC WHO Clinical Characterisation Protocol: prospective observational cohort study. BMJ.

[29] Liu Y, Yan LM, Wan L (2020). Viral dynamics in mild and severe cases of COVID-19. Lancet Infect Dis.

[30] Gaillard F, Ismael S, Sannier A (2020). Tubuloreticular inclusions in COVID-19-related collapsing glomerulopathy. Kidney Int.

