# Supplementary material for: Proteinuria in COVID-19: prevalence, characterization and prognostic role
Source: J Nephrol. 2021 Jan 23;34(2):355–64. doi: 10.1007/s40620-020-00931-w (PMC7823174; doi:10.1007/s40620-020-00931-w)

**Proteinuria in COVID-19: prevalence, characterization and prognostic role: Supplementary Appendix**

**Huart J, MD^1,2^, Bouquegneau A, MD^1^, Lutteri L, EuSpLM^3^, Erpicum P, MD, PhD^1,2^, Grosch S, MD^1^, Résimont G, MD^1^, Wiesen P, MD^4^, Bovy C, MD, PhD^1^, Krzesinski JM, MD, PhD^1,2^, Thys M^5^, Lambermont B, MD, PhD^4^, Misset B, MD, PhD^4^, Pottel H, PhD^6^, Mariat C, MD, PhD^7^, Cavalier E, EuSpLM, PhD^3^, Burtey S, MD, PhD^8^, Jouret F, MD, PhD^1,2^, Delanaye P, MD, PhD^1,9^**

**^1^Department of Nephrology-Dialysis-Transplantation, CHU Sart Tilman, University of Liège (ULg CHU), Liège, Belgium**

**^2^Groupe Interdisciplinaire de Géno-protéomique Appliquée, Cardiovascular Sciences, University of Liège, Liège, Belgium**

**^3^Department of Clinical Chemistry, CHU Sart Tilman, University of Liège (CHU ULg), Liège, Belgium**

**^4^Department of Intensive Care, CHU Sart Tilman, University of Liège (CHU ULg), Liège, Belgium**

**^5^ Department of Medico-Economic Information, CHU Sart Tilman, University of Liège (CHU ULg), Liège, Belgium**

**^6^Department of Public Health and Primary Care, KU Leuven Campus Kulak Kortrijk, Kortrijk, Belgium**

**^7^Nephrology, Dialysis and Renal Transplantation Department, Hôpital Nord, CHU de Saint-Etienne, Jean Monnet University, COMUE Université de Lyon, Lyon, France**

**^8^Center of Nephrology and Renal Transplantation, Public Assistance of the Hospitals of Marseille, Marseille, France; Aix-Marseille University, INSERM, INRA, C2VN, Marseille, France**

**^9^Department of Nephrology-Dialysis-Apheresis, Hopital Universitaire Caremeau, Nimes, France**

**Corresponding author: Pierre Delanaye, Service de Dialyse, CHU Sart Tilman, 4000 Liège, Belgium, Phone: ++3243667111, Fax: ++3243667205, pierre_delanaye@yahoo.fr**

**Table of contents**

**Table S1:** Clinical and biological characteristics of the patients included or not in the analysis (p.3)

**Table S2:** Clinical and biological characteristics of the patients at D0 according to tertiles of urinary β_2_-microglobulin at D0 (p.4)

**Table S3:** Clinical and biological characteristics of the patients at D0 according to mortality status (p.5)

**Table S4:** Urine parameters at D0 according to mortality status in different subgroups (p.6)

**Figure S1:** Flowchart of patients’ inclusion (p.7)

**Table S1: Clinical and biological characteristics of the patients included or not in the analysis**

|  | **Included (n=153)** | **Not-included (n=72)** | **P values** |
| --- | --- | --- | --- |
| **CLINICAL** |  |  |  |
| Age (years) | 70 [58;81] | 72 [59;82] | ns |
| Women (n=153) (%) | 39 | 54 | 0.0348 |
| Weight (kg) | 80±17 | 74±17 | ns |
| Height (m) | 1.70 [1.62;1.78] | 1.68 [1.61;1.74] | ns |
| Body Mass index (kg/m²) | 28 [24;31] | 26 [22;30] | ns |
|  |  |  |  |
|  |  |  |  |
| In-hospital mortality (%)  Time to death (day) | 15  11 [6;15] | 38  3 [2;10] | 0.0002  0.0009 |
| Intensive Care Unit (%) | 25 | 14 | ns |
| **BIOLOGICAL** |  |  |  |
| Creatinine (admission) (mg/dL)  eGFR (mL/min/1.73m²) | 1.00 [0.83;1.33]  69 [50 ;87] | 1.05 [0.73;1.97]  63 [28 ;86] | ns  ns |
| C-reactive protein (mg/L) | 81 [38;155] | 87 [36;152] | ns |
| Procalcitonin (µg/L) | 0.14 [0.07;0.34] | 0.15 [0.06;0.51] | ns |
| Leucocytes count (/mm³) | 6180 [4650;9060] | 7100 [4660;9720] | ns |
| Lymphocytes count (/mm³) | 860 [655;1155] | 1000 [670;1480] | 0.0145 |
| Hemoglobin (g/dL) | 12.3 [11.2;13.6] | 13.0 [11.8;14.1] | ns |
| Platelet (x1000/mm³) | 230 [165;301] | 194 [163;256] | ns |
| Lactate Dehydrogenase (U/L) | 342 [259;443] | 324 [247;433] | ns |
| D-Dimer (µg/L) | 1009 [715;1878] | 926 [526;2200] | ns |
| Albumin (g/L) | 34±5 | 35±5 | ns |
| Potassium (mmol/L) | 4.10±0.47 | 3.99±0.56 | ns |
| Sodium (mmol/L) | 139 [137;142] | 141 [137;143] | ns |
| Calcium (mmol/L) | 2.10 [2.01;2.20] | 2.19 [2.05;2.35] | 0.0034 |
| Bicarbonates (mmol/L) | 25.5 [23.1;27.6] | 23.2 [21.2;26.4] | 0.0039 |

Ns: not significant

eGFR: estimated glomerular filtration rate

**Table S2: Clinical and biological characteristics of the patients at D0 according to tertiles of urinary β_2_-microglobulin at D0**

|  | **Tertile 1**  **<0.74 mg/L**  **(n=31)** | **Tertile 2**  **0.74-5.18 mg/L**  **(n=31)** | **Tertile 3**  **>5.18 mg/L**  **(n=32)** | **Kruskal Wallis test**  **(or exact Chi² test)** |
| --- | --- | --- | --- | --- |
| **CLINICAL** |  |  |  |  |
| Age (years) | 66 [58;79] | 73 [65;81] | 74 [54;83] | ns |
| Women (%) | 45 | 39 | 38 | ns |
| Weight (kg) | 76 [65;93] | 76 [70;90] | 72 [67;88] | ns |
| Height (m) | 1.67 [1.61;1.75] | 1.69 [1.65;1.75] | 1.72 [1.66;1.80] | ns |
| Body Mass index (kg/m²) | 28 [24;31] | 28 [25;29] | 26 [22;29] | ns |
| Medical history  Hypertension (%)  Diabetes (%)  Chronic kidney disease (%)  Active cancer (%)  Active smoking (%) | 61  29  16  6  3 | 74  29  13  6  10 | 56  31  31  9  0 | ns  ns  ns  ns  ns |
| Intensive Care Unit (%) | 0 | 16 | 19^$^ | 0.04 |
| Severe cases (%) | 65 | 84 | 88 | 0.03 |
| **BIOLOGICAL** |  |  |  |  |
| Creatinine (D0) (mg/dL)  eGFR (mL/min/1.73m²) | 0.87 [0.71;1.05]  82 [62;93] | 0.92 [0.76;1.28]  71 [54;86] | 0.97 [0.77;1.33]  72 [40;89] | ns  ns |
| C-reactive protein (mg/L) | 63 [19;117] | 66 [31;156] | 79 [38;163] | ns |
| Procalcitonin (µg/L) | 0.12 [0.05;0.23] | 0.11 [0.08;0.43] | 0.16 [0.06;0.35] | ns |
| Leucocytes count (/mm³) | 6250 [4583;7660] | 6440 [4580;11010] | 5870 [4660;6970] | ns |
| Lymphocytes count (/mm³) | 990 [703;1382] | 930 [755;1340] | 815 [670;1070] | ns |
| Hemoglobin (g/dL) | 12.2 [10.9;13.2] | 12.2 [10.9;13.4] | 11.9 [11.1;13.1] | ns |
| Platelet (x1000/mm³) | 281 [206;352] | 234 [164;292] | 197 [162;278] | ns |
| Lactate Dehydrogenase (U/L) | 324 [240;408] | 348 [270;446] | 332 [261;412] | ns |
| D-Dimer (µg/L) | 986 [702;1610] | 965 [691;1494] | 1130 [741;2498] | ns |
| Albumin (g/L) | 34 [31;43] | 35 [32;38] | 35 [29;37] | ns |
| Potassium (mmol/L) | 4.11 [3.82;4.53] | 4.16 [3.84;4.59] | 4.03 [3.69;4.33] | ns |
| Sodium (mmol/L) | 141 [138;143] | 139 [136;142] | 139 [136;143] | ns |
| Calcium (mmol/L) | 2.20 [2.11;2.29] | 2.07 [1.98;2.19]^$^ | 2.07 [2.00;2.11]^$^ | 0.003 |
| Bicarbonates (mmol/L) | 24.1 [23.0;27.0] | 23.9 [22.5;25.6]^*^ | 26.1 [23.6;28.6] | 0.045 |

Ns: not significant

eGFR: estimated glomerular filtration rate

Dunn’s post-hoc test p<0.05: * proteinuria category 3, $ with proteinuria category 1

Chi² test for categorical variables with Bonferroni correction: * with proteinuria category 3

**Table S3: Clinical and biological characteristics of the patients at D0 according to mortality status**

|  | **Alive (n=126)** | **Dead (n=27)** | **Mann Whitney test or Chi² test** |
| --- | --- | --- | --- |
| **CLINICAL** |  |  |  |
| Age (years) | 68 [58;80] | 78 [70;85] | 0.02 |
| Women (%) | 43 | 19 | 0.02 |
| Weight (kg) (n=130) | 78 [69;92] | 77 [66;93] | ns |
| Height (m) (n=125) | 1.70 [1.62;1.78] | 1.71 [1.67;1.79] | ns |
| Body Mass index (kg/m²) (n=120) | 28 [24;30] | 27 [23;31] | ns |
| Medical history  Hypertension (%)  Diabetes (%)  Chronic kidney disease (%)  Active cancer (%)  Active smoking (%) | 58  26  14  8  4 | 63  37  30  22  7 | ns  ns  0.04  0.03  ns |
| Intensive Care Unit (%) | 20 | 30 | ns |
| Severe case (%) | 79 | 93 | ns |
| Thoracic CT-Scanner staging  Normal  Minor (<10%)  Mild (10-50%)  Severe (>50%) | 13  9  60  18 | 22  0  63  15 | ns  ns  ns  ns |
| **BLOOD PARAMETERS** |  |  |  |
| Creatinine (D0) (mg/dL)  eGFR (mL/min/1.73m²) | 0.91 [0.75;1.08]  80 [59;92] | 1.15 [0.91;1.52]  61 [43;85] | 0.02  0.03 |
| C-reactive protein (mg/L) | 78 [30;147] | 124 [63;182] | 0.02 |
| Procalcitonin (µg/L) | 0.13 [0.06;0.31] | 0.19 [0.09;0.46] | ns |
| Leucocytes count (/mm³) | 6115 [4630;8370] | 6870 [5050;9370] | ns |
| Lymphocytes count (/mm³) | 870 [668-1195] | 820 [420;1020] | ns |
| Hemoglobin (g/dL) | 12.6 [11.2;13.7] | 11.7 [11.0;12.9] | ns |
| Platelet (x1000/mm³) | 241 [178;316] | 173 [144;238] | 0.003 |
| Lactate Dehydrogenase (U/L) | 335 [257;442] | 362 [272;505] | ns |
| D-Dimer (µg/L) | 985 [709;1629] | 1793 [675 ;2969] | ns |
| Albumin (g/L) | 34 [31 ;38] | 33 [29;36] | ns |
| Potassium (mmol/L) | 4.06 [3.80;4.31] | 4.36 [3.69;4.72] | ns |
| Sodium (mmol/L) | 140 [137;142] | 139 [136;142] | ns |
| Calcium (mmol/L) | 2.10 [2.02;2.20] | 2.09 [1.97;2.29] | ns |
| Bicarbonates (mmol/L) | 25.0 [23.1;27.4] | 25.7 [22.6;29.0] | ns |
| **URINE PARAMETERS** |  |  |  |
| Proteinuria | 390 [218;747] | 731 [486;1277] | 0.0007 |
| Urine α_1_-microglobulin | 49 [24;89] | 132 [48;182] | 0.0003 |
| Urine β_2_-microglobulin (n=94) | 0.97 [0.30;7.58] | 11.22 [4.17;67.80] | 0.005 |
| Urinary catheter | 23 | 37 | ns |
| Hematuria (number per field) | 0 [0;9] | 7 [0;36] | 0.03 |

Ns: not significant

eGFR: estimated glomerular filtration rate

**Table S4: Urine parameters at D0 according to mortality status in different subgroups**

|  | **Alive** | **Dead** | **Mann Whitney test or Chi² test** |
| --- | --- | --- | --- |
| **Cohort with decreased GFR at D0 (eGFR_age_)** | **n=22** | **n=9** |  |
| Proteinuria (mg/g) | 429 [303;917] | 557 [441;1260] | ns |
| Urine α_1_-microglobulin (mg/g) | 72 [45;141] | 134 [81;173] | ns |
| Urine β_2_-microglobulin (n=14+7) (mg/L) | 4.12 [0.96;15.10] | 23.00 [4.27;63.38] | ns |
| Urinary catheter (%) | 41 | 44 | ns |
| Hematuria (number per field) | 3 [0;18] | 0 [0;4] | ns |
| **Cohort with normal GFR at D0 (eGFR_age_)** | **n=104** | **n=18** |  |
| Proteinuria (mg/g) | 384 [192;744] | 761 [522;1392] | 0.002 |
| Urine α_1_-microglobulin (mg/g) | 40 [23;84] | 132 [40;205] | 0.001 |
| Urine β_2_-microglobulin (n=62+11) (mg/L) | 0.84 [0.27;6.11] | 8.44 [2.43;56.50] | 0.03 |
| Urinary catheter (%) | 4 | 61 | <0.0001 |
| Hematuria (number per field) | 0 [0;8] | 26 [0;165] | 0.0007 |
| **Cohort with urinary catheter** | **n=24** | **n=15** |  |
| Proteinuria (mg/g) | 625 [465;1107] | 701 [472;1019] | ns |
| Urine α_1_-microglobulin (mg/g) | 73 [39;121] | 129 [48;161] | ns |
| Urine β_2_-microglobulin (n=11+6) (mg/L) | 3.41 [0.79;24.04] | 11.22 [4.61;14.10] | ns |
| Hematuria (number per field) | 22 [9;88] | 17 [0;233] | ns |
| **Cohort without urinary catheter** | **n=102** | **n=12** |  |
| Proteinuria (mg/g) | 320 [170;618] | 1067 [540;1490] | 0.0005 |
| Urine α_1_-microglobulin (mg/g) | 42 [23;81] | 137 [60;227] | 0.002 |
| Urine β_2_-microglobulin (n=65+12) (mg/L) | 0.91 [0.29;7.43] | 13.79 [2.99;69.20] | 0.02 |
| Hematuria (number per field) | 0 [0;0] | 0 [0;22] | ns |

Ns: not significant

eGFR: estimated glomerular filtration rate

**Figure S1: Flowchart of patients’ inclusion**


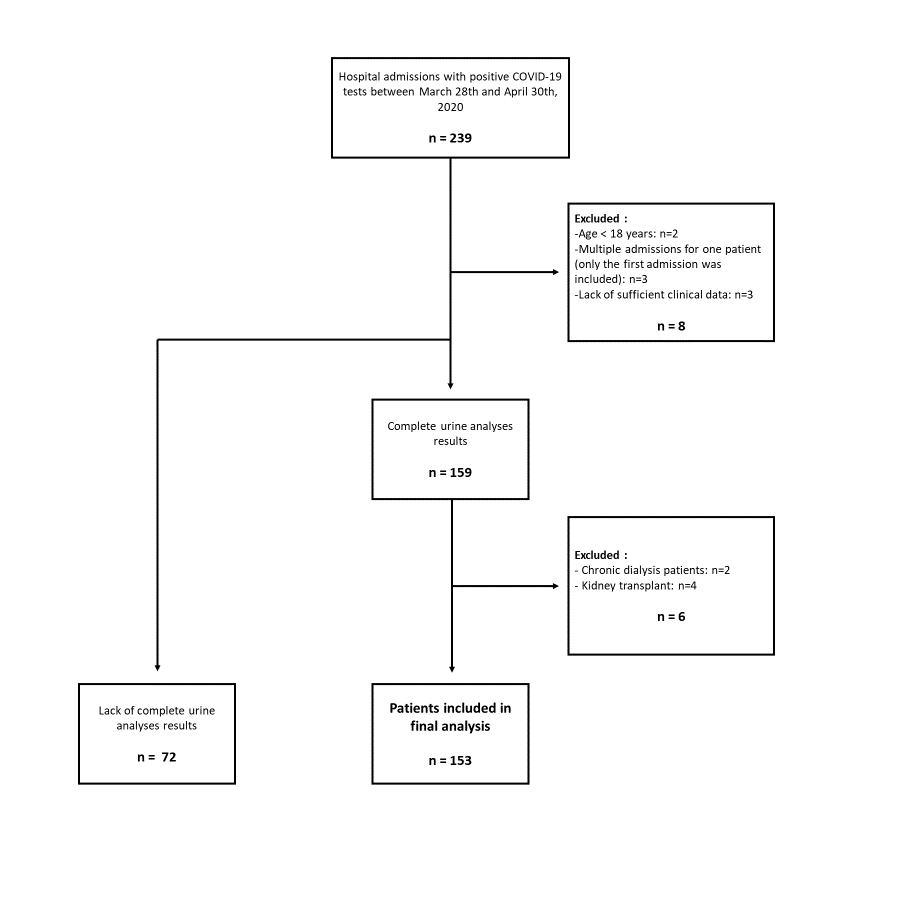

Supplement: Supplementary file 1 — Supplementary material 1 (DOCX 55 kb) [file 40620_2020_931_MOESM1_ESM.docx]
